# Genetically determined fungal pathogen tolerance and soil variation influence ectomycorrhizal traits of loblolly pine

**DOI:** 10.1002/ece3.4355

**Published:** 2018-09-05

**Authors:** Bridget J. Piculell, Lori G. Eckhardt, Jason D. Hoeksema

**Affiliations:** ^1^ Department of Biology University of Mississippi University Mississippi; ^2^ Department of Biology College of Charleston Charleston South Carolina; ^3^ School of Forestry and Wildlife Sciences Auburn University Auburn Alabama

**Keywords:** fusiform rust disease, indirect selection, loblolly pine (*Pinus taeda* L.), mycorrhizal fungi, pine decline, trait correlations

## Abstract

Selection on genetically correlated traits within species can create indirect effects on one trait by selection on another. The consequences of these trait correlations are of interest because they may influence how suites of traits within species evolve under differing selection pressures, both natural and artificial. By utilizing genetic families of loblolly pine either tolerant (t) or susceptible (s) to two different suites of pathogenic fungi responsible for causing either pine decline or fusiform rust disease, we investigated trait variation and trait correlations within loblolly pine (*Pinus taeda* L.) by determining how ectomycorrhizal (EM) colonization relates to pathogen susceptibility. We detected interactions between susceptibility to pathogenic fungi and soil inoculation source on loblolly pine compatibility with the EM fungi *Thelephora*, and on relative growth rate of loblolly pine. Additionally, we detected spatial variation in the loblolly pine–EM fungi interaction, and found that variation in colonization rates by some members of the EM community is not dictated by genetic variation in the host plant but rather soil inoculation source alone. The work presented here illustrates the potential for indirect selection on compatibility with symbiotic EM fungi as a result of selection for resistance to fungal pathogens. Additionally, we present evidence that the host plant does not have a single “mycorrhizal trait” governing interactions with all EM fungi, but rather that it can interact with different fungal taxa independently. *Synthesis*. An understanding of the genetic architecture of essential traits in focal species is crucial if we are to anticipate and manage the results of natural and artificial selection. As demonstrated here, an essential but often overlooked symbiosis (that between plants and mycorrhizal fungi) may be indirectly influenced by directed selection on the host plant.

## 1 INTRODUCTION

Selection on genetically correlated traits within species can create indirect effects on one trait by selection on another. Such genetic correlations between traits can determine how populations evolve under multiple, conflicting selection pressures (Ridenhour, 2005; Whitlock, Phillips, Moore, & Tonsor, 1995). They can lead to maladaptation of one trait driven by strong selective pressures on another, as well as potential facilitation by one trait on another's evolution (Futuyma, 2010; Lynch, 1999). These indirect effects could constrain the adaptation of species to their environment, and to each other, as multiple selection pressures acting simultaneously on different traits of an organism create conflicts as to the ideal evolutionary trajectory of a population (Griswold & Whitlock, 2003; Lynch, 1999; Ridenhour, 2005; Wade, 2001). An understanding of the correlations among traits in populations is thus important when assessing the ability of a population to persist in or adapt to its natural environment, and also when considering how a population may respond to artificial selection. In the laboratory experiment described here, we assessed the degree of correlation among traits mediating the interaction of loblolly pine (*Pinus taeda* L.) with both pathogenic and mycorrhizal fungi, and the impact of environmental variation on those interactions.

Mycorrhizal fungi are common symbionts of most plants, deriving mineral nutrients from the soil and transferring them to the host, while the host provides carbohydrates to the fungi. Mycorrhizal fungi also have been shown to affect essential host traits such as drought tolerance, and to alter competitive interactions within and among plant species (Bennet & Cahill, 2016; Gehring, Sthultz, Flores‐Renteria, Whipple, & Whitham, 2017; Sebastiana et al., 2018; Smith & Read, 2008). It has been estimated that from 6,000 (Brundrett, 2002) to as many as 20,000 (Rinaldi, Comandini, & Kuyper, 2008) different species of fungi form a particular type of mycorrhizae, ectomycorrhizae (EM). The EM fungi include both host specialists and generalists, with host plants capable of simultaneous interaction with several to hundreds of different fungal partners, and most EM fungi having the ability to associate with more than one host species (reviewed by Smith & Read, 2008).

Given the multi‐partner patterns that we see in mycorrhizal interactions, it has been hypothesized that coevolution between the interacting species is too diffuse to be ongoing, and when it did occur it was early in the evolution of the symbiosis (Cairney, 2000). There is, however, evidence to suggest the potential for more recent coevolution in mycorrhizal symbioses, although relatively few of the relevant experiments have been performed (Hoeksema, 2010). For example, in a study investigating the influence of soil and EM community on assisted migration of Douglas‐fir (*Pseudotsuga* menziesii), Kranabetter (2005) found that as the home EM community of transplanted hosts diverged from that of the local population, host productivity declined, indicating local adaptation with site‐adapted EM communities. Such results are intriguing, but so few such studies have been conducted that it is difficult to generalize, and there is much we do not know about ongoing coevolution in mycorrhizal interactions. For instance, we know little about how evolution of mycorrhizal symbiosis traits is influenced by their genetic architecture and genetic correlations with other traits, including those governing interactions with additional species outside the symbiosis.

In addition to mycorrhizal fungi, loblolly pine populations interact regularly with fungal pathogens, such as those causing fusiform rust disease (*Cronartium quercuum* (Berk) Miyabe ex Shirai f. sp. *fusiforme*) and those associated with the pine decline complex. The pine decline complex is associated with several abiotic and biotic factors, including *Leptographium* and *Grosmannia* pathogenic fungal species, with symptoms including short, chlorotic needles, and thinned crowns (review in Eckhardt, Weber, Menard, Jones, & Hess, 2007). Fusiform rust is a disease that can deform or even kill pines (especially *Pinus taeda* L. and *P. elliottii* Engelm.). These fungal pathogens have had negative economic and environmental impacts on both natural and agriculturally managed loblolly pine stands, causing substantial damage yearly. Several studies have shown variation among loblolly pine genetic families in their susceptibility to both fusiform rust disease (Isik et al., 2008; Li, McKeand, & Weir, 1999) and pine decline (Singh, Anderson, & Eckhardt, 2014). Studies such as these demonstrate the potential for evolution of pathogen tolerance in response to artificial and natural selection, but what we do not know is how selection on these traits might influence other important traits of loblolly pine, such as those mediating interactions with other species or communities, such as the soil borne mycorrhizal fungal community.

Several studies examining artificially selected crop plants suggest that traits mediating mycorrhizal associations of plants may be genetically correlated with other traits. For example, Zhu, Smith, Barritt, and Smith (2001) found that modern cultivars of wheat had reduced mycorrhizal colonization compared to older cultivars, while Bryla and Koide (1990) found modern cultivars of tomato (*Lycopersicon esculentum* Mill) to show greater vegetative and reproductive responsiveness to mycorrhizal colonization than wild strains. These studies suggest that although artificial selection in these plants was for other, agriculturally relevant traits, the association with mycorrhizal fungi was indirectly affected. Studies of pinyon pine (*Pinus edulis*) have shown that trees differing genetically in tolerance to insect pests also host different EM fungal communities, whether or not herbivory has occurred (Sthultz, Whitham, Kennedy, Deckert, & Gehring, 2009). Additionally, it has been found that the EM fungal community of insect‐susceptible and insect‐resistant trees responds differently to drought conditions (Gehring et al., 2014). Work on the genetic map of poplars (*Populus trichocarpa*) has revealed a quantitative trait locus associated with compatibility with a particular EM fungal species that maps near a linkage group determined to be involved in tolerance to rust fungi (Tagu, Lapeyrie, & Martin, 2002), suggesting that at least one pleiotropic locus may be influencing both traits. Thus, selection for tolerance in poplars to rust infection by the fungus *Melampsora larici‐populina* could affect the evolution of traits governing mycorrhizal colonization. Similar results may be expected in other plants, including loblolly pine. These results may be more interesting when considered in a more complete community context, including the diverse suite of mycorrhizal fungi that typically associate with pines.

Furthermore, the outcomes of species interactions may vary spatially depending on variation in the biotic and abiotic contexts in which they occur. This has the potential to create a geographic “selection mosaic,” wherein populations of interacting species vary in the selection pressures that each species exerts on each other's traits (Thompson, 1994, 2005). Loblolly pine occurs nearly continuously across the southeastern United States in both natural and agricultural stands (Schultz, 1997). This broad range makes incorporation of site variation an important consideration when studying the interaction outcomes of this system. By analyzing the coevolutionary interaction (G × G) between loblolly pine and mycorrhizal fungi within different environments (G × G × E), we may be able to better understand the effects of natural and artificial selection on this complex and pervasive mutualism.

Here we report the results of a growth chamber experiment designed to investigate genetic variation in traits and trait correlations within loblolly pine by investigating how patterns of EM colonization correspond to pathogen susceptibility and fungal community inoculation source. The experiment utilized multiple genetic families of loblolly pine previously determined to be either tolerant or susceptible to two different suites of pathogenic fungi responsible for causing either pine decline or fusiform rust disease, and exposed those families to different mycorrhizal fungal inoculation regimes. We allowed pathogen‐tolerant and susceptible seedlings access to whole soil fungal communities from three different locations within the natural range of loblolly pine. By studying genotypes that vary in susceptibility to one of the selection pressures shaping populations (fungal pathogens), we may be able to understand how indirect selection may be driving evolution in other traits, such as compatibility with particular mycorrhizal fungi. Examination of mycorrhizal traits in loblolly pine families that vary in susceptibility to fungal pathogens, but have not been exposed to the pathogens, will also help disentangle patterns seen in field, where it is difficult to establish the mechanism behind observed correlations between traits. For example, correlations between mycorrhizal traits and pathogen tolerance could be a product of genes influencing both traits directly, or it could be that fungal pathogens induce a response in the host plant that affects its association with mycorrhizal fungi. Additionally, by utilizing soil from multiple locations within the range of loblolly pine, this field soil inoculation experiment allows exploration of variation in EM fungal community composition and tests the potential for host genotypes to be expressed differently in different biotic environments.

Specifically, we aimed to explore genetic variation, trait correlations, and geographic variation in the loblolly pine–mycorrhizal fungi interaction by answering these questions:

1. Do different soil inoculation sources within the natural range of loblolly pine yield different mycorrhizal fungal communities?
2. Do individual EM fungi respond differently to host genetic variation in pathogen tolerance and does this response depend on origin of the fungal community?
3. How do other traits (host plant relative growth rate [RGR], root–shoot ratio, and number of root tips colonized by EM fungi per cm root) respond to host genetic variation in pathogen tolerance and does this depend on origin of the fungal community?

## 2 MATERIALS AND METHODS

### 2.1 Seedlings and soil

Loblolly pine seeds were obtained from open‐pollinated families that fell into one of four genetic categories: pine decline tolerant (PDt: 4 families), pine decline susceptible (PDs, 4 families), fusiform rust tolerant (FRt, 4 families), and fusiform rust susceptible (FRs, 6 families). These categories were determined in previous pathogen inoculation trials, and tolerant and susceptible families were chosen from the upper and lower ends of the genetic distribution of tolerance to each pathogen (Singh et al., 2014, L. G. Eckhardt, unpublished data). Seeds were surface sterilized with 5% bleach and cold stratified for 40 days, after which they were planted in trays with sterile peatmoss/perlite potting soil (Metro‐Mix 360) and kept in a Conviron Model CMP6050 environmental growth chamber at 26°C with a 14‐hr photoperiod (~302 μmol m^−2^ s^−1^), receiving weekly deionized water sufficient to completely soak the soil. Six weeks after planting, seedlings from each family were transplanted to bleach‐sterilized Ray Leach cone‐tainers (SC10, 164 ml; Stuewe & Sons Inc., Tangent, OR).

### 2.2 Field soil inoculation

**Figure 1:**
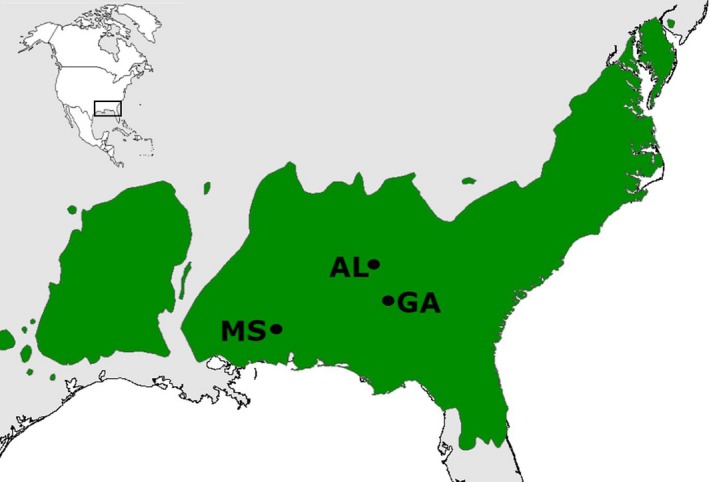
Distribution of loblolly pine native range across the southeastern United States, shown in green. Field soil sampling locations in Mississippi (MS, N 31.14785°W −88.48038), Alabama (AL, N 32.49904°W −85.57245), and Georgia (GA, N 32.003°W −84.981) shown in blue

Field soil was collected from three loblolly pine stands located centrally within the natural range of loblolly pine (Stateline MS, N 31.14785°W −88.48038; Ray 9 GA, N 32.003°W −84.981; Tuskegee AL, N 32.49904°W −85.57245) (Figure 1). Soil from each site was separately homogenized and sifted over a 1‐cm sieve to remove large debris. Unsterilized samples of each soil were set aside for inoculation of pots with biotic communities from each site, while the majority of soil was mixed in equal parts with soil from the other two sites, and autoclaved at 121°C for 1 hr to sterilize for use as a base soil in all treatments. Three soil inoculation treatments were created by inoculating a subset of the base soil with unsterilized soil from each of the three locations, resulting in approximately ¼ of the soil being comprised of unsterilized soil. This method allowed us to reduce the influence of variation in soil chemical and physical properties among the different sites, while conducting a bioassay of the spore‐bank EM fungi from three local EM fungal and other microbiotic communities.

Seedlings representing each of the four tolerance types (pine decline tolerant, PDt: pine decline susceptible, PDs; fusiform rust tolerant, FRt; fusiform rust susceptible FRs) were transplanted into pots containing the three different field soil (Mississippi, MS; Alabama, AL; Georgia, GA) treatments (PDt: MS *n* = 200, AL *n* = 200, GA *n* = 194; PDs: MS *n* = 200, AL *n* = 200, GA *n* = 200; FRt: MS *n* = 200, AL *n* = 200, GA *n* = 197; FRs: MS *n* = 259, AL *n* = 259, GA *n* = 254). Transplant mortality was assessed 55 days after initial planting: 586 of the 2,563 seedlings (22.9%) had died, with no significant difference among soil types or plant family categories. High seedling mortality was attributed to transplanting and transportation. Additional mortality occurred throughout the 22‐week growth period, with final replicate numbers in each genetic category as follows: PDt: MS *n* = 50, AL *n* = 48, GA *n* = 23; PDs: MS *n* = 43, AL *n* = 50, GA *n* = 41; FRt: MS *n* = 42, AL *n* = 52, GA *n* = 37; FRs: MS *n* = 54, AL *n* = 63, GA *n* = 55 (Table 1). Seedlings were kept in a growth chamber at 26°C with a 14‐hr photoperiod (~302 μmol m^−2^ s^−1^), receiving a weekly watering sufficient to completely soak the soil. Seedlings in cone‐tainers were randomized in trays within each soil type (to avoid contamination between soils) at the beginning of the experiment and then re‐randomized after 10 weeks of growth. Each tray contained seedlings of the same inoculation treatment to avoid splash contamination during watering. The locations of the trays in the growth chamber were also randomized. Seedling height was measured at planting (Ht1) and upon harvest (Ht2), which took place 22 weeks after planting in treatment soil; this allowed for calculation of RGR of height (RGR = (ln(Ht2) − ln(Ht1))/(no. days of growth)). All plants were assayed for mycorrhizal fungal colonization characteristics including colonization intensity (number of root tips colonized per cm root) and abundance of different EM morphotypes (Table 2), which were based on characteristics visible under a dissecting microscope, including color, texture, and abundance of emanating hyphae and rhizomorphs (Agerer, 2001). Root length was estimated using the grid‐line intersect method (Newman, 1966). Above‐ and belowground portions of each plant were dried at 60°C and root and shoot dry biomass were determined.

**Table 1:** Number of *Pinus taeda* seedlings planted in field soils (MS, Mississippi; AL, Alabama; and GA, Georgia) from each loblolly family pathogen resistance category (FRt, fusiform rust tolerant; FRs, fusiform rust susceptible; PDt, pine decline tolerant; PDs, pine decline susceptible). Total *n* = 558

Category	MS	AL	GA
FRt	42	52	37
FRs	54	63	55
PDt	50	48	23
PDs	43	50	41
Total	189	213	156

**Table 2:** Morphological characteristics of the four dominant ectomycorrhizal fungal morphotypes found on loblolly pine (*Pinus taeda* L.) seedlings

Fungal morphotype	Description
Rhizopogon	White/pale in color, densely colonized (often occurring in large coralloid clusters), with emanating rhizomorphs
Cenococcum	Dark black in color, usually solitary (not clustered), but still numerous. Copious dense emanating hyphae
Wilcoxina	Noticeably darker brown than root, with slight constriction at base, often with white/clear tip. Color and width variation along length. Very long with no branching. Sometimes solitary but usually found in patches of multiple colonized tips
Thelephora	Orange‐light brown in color, slightly lighter at tips. Sometimes very long with no branching, but occasionally with single shorter side branch. Narrow at base, but widening noticeably towards center

### 2.3 Statistical analyses

2.3.1

All analyses were done with R statistical software, version 3.2.1 (R Core Team, 2015). To determine if EM fungal morphotype composition differed among soil inoculation sources (Question 1), permutational MANOVA was performed using the *adonis* function from the *vegan* package in R (Oksanen et al., 2015), with the response variable being a Bray–Curtis dissimilarity matrix generated using the *vegdist* function from the *vegan* package (Oksanen et al., 2015), and the predictor variables Soil (MS, GA, AL), Genetic Category (pine decline tolerant: PDt; pine decline susceptible: PDs; fusiform rust tolerant: FRt; and fusiform rust susceptible: FRs), and Soil × Genetic Category interaction, with loblolly seed family included as a random effect nested within Genetic Category. Multivariate dispersion was checked using the *betadisper* function in the *vegan* package (Oksanen et al., 2017) and was found to vary among soil types (*F*_2,558_ = 7.16, *p *=* *0.0008); however, given the large sample sizes and visual confirmation of community differences, we treated this as an acceptable violation. The fungal morphotypes included in this and all subsequent analyses were only those four morphotypes that comprised >5% of the total number of root tips colonized in each soil type. In removing the rarer species, we follow recommendations by some statisticians (e.g., McCune, Grace, & Urban, 2002), who have argued that for testing effects of experimental variables on multivariate community composition, deleting rare species may be desirable because it can reduce noise in the data (and thus improve detection of relationships) without losing much information.

To answer Questions 2 and 3, we analyzed separate univariate Type III ANOVA models for each response variable using the *lmer* function from the *lme4* package (Bates et al., 2015) in R, and the *anova* function from the core *stats* package for each of the seven response variables measured: RGR, number of colonized root tips per centimeter root (tips/cm, square root transformed to achieve normality of residuals), root:shoot ratio, and percentage of total colonized root tips that were colonized by each of the four dominant fungal morphotypes. The fixed factors in each model were Soil (MS, GA, AL), Genetic Category (pine decline tolerant: PDt; pine decline susceptible: PDs; fusiform rust tolerant: FRt; and fusiform rust susceptible: FRs), and Soil × Genetic Category interaction, with loblolly seed family included as a random effect within Genetic Category. When the Soil × Genetic Category interaction was found to be significant, we performed pre‐determined contrasts (PDt vs. PDs, and FRt vs. FRs) for each response variable to determine differences between plants tolerant and susceptible to each of the fungal pathogens within and between soils, using the *glht* function from the *multcomp* package (Hothorn et al. 2008). When either Soil or Genetic Category was significant without interaction, we used the *difflsmeans* function from the *lmerTest* package (Kuznetsova et al. 2016) to separate means. Comparisons were not made across pathogen categories (for example, comparison of pine decline‐tolerant plants and fusiform rust‐tolerant plants) because the seed families obtained were only categorized as either resistant or susceptible to one or the other fungal pathogen. Additionally, we analyzed the relationship between RGR and the five EM colonization measurements: percentage of total colonized root tips that were colonized by each of the four dominant fungal morphotypes, and number of colonized root tips per centimeter root (tips/cm, square root transformed to achieve normality of residuals). This was achieved by analyzing separate univariate Type III ANOVA models using the *lmer* function from the *lme4* package (Bates et al., 2015) in R, and the *anova* function from the core *stats* package. RGR was the response variable, with separate models for each fungal predictor, and loblolly seed family within Genetic Category and Soil included as random effects.

## 3 RESULTS

### 3.1 Question 1: Do different locations within the natural range of loblolly pine yield different mycorrhizal fungal communities?

**Figure 2:**
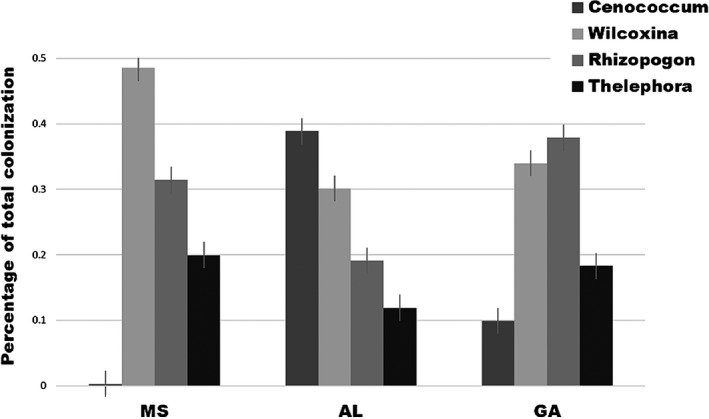
Soil fungal community composition in the three field soil locations, Mississippi (MS), Alabama (AL), and Georgia (GA). We found the three different soil inoculation sources, while containing the same four dominant fungal morphotypes, *Rhizopogon*,* Cenococcum*,* Wilcoxina*, and *Thelephora*, each to have a different composition of those fungi (*F*_2,560_ = 36.754, *p *= 0.01, *R* ^2^ = 0.12). All data are presented as means ± *SE*

We found the three different soil inoculation sources, while containing the same four dominant fungal morphotypes—*Rhizopogon*,* Cenococcum*,* Wilcoxina*, and *Thelephora*—each to have a significantly different composition of those fungi (*F*_2,560_ = 36.75, *p *=* *0.01, *R* ^2^ = 0.12) (Figure 2). Across the three soil inoculation sources (MS, AL, GA), four morphotypes of fungi were identified as the most dominant root tip colonizers of all seedlings (measured as percentage of total root tips colonized), *Rhizopogon*,* Cenococcum*,* Wilcoxina*, and *Thelephora* (see Table 2 for morphotype descriptions).

### 3.2 Question 2: Do individual EM fungi respond differently to host genetic variation in pathogen tolerance and does this genetic variation depend on origin of the fungal community?

**Figure 3:**
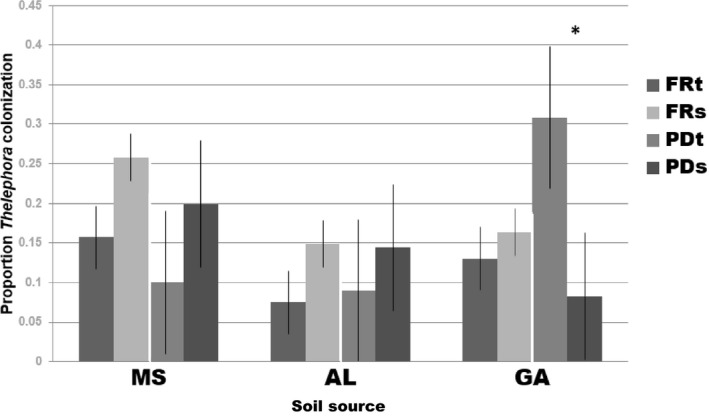
The influence of pathogen resistance category (FRt, fusiform rust tolerant; FRs, fusiform rust susceptible; PDt, pine decline tolerant; PDs, pine decline susceptible) on proportion of root tips colonized by *Thelephora* varied among soil inoculation sources (MS, Mississippi; AL, Alabama; and GA, Georgia), (Category × Soil interaction: *F*_6,537_ = 3.74, *p* = 0.001). Pine decline‐tolerant plants had significantly greater colonization by *Thelephora* in Georgia soil, compared to pine decline‐susceptible plants (*p = *2.0e‐4), but the two categories did not differ in either Mississippi or Alabama soils*;* fusiform rust‐tolerant and susceptible plants did not differ in *Thelephora* colonization

Of the four dominant morphotypes, only *Thelephora* was influenced by factors other than soil inoculation source. We found that the influence of Genetic Category on abundance of *Thelephora* varied among soils, (Genetic Category × Soil interaction: *F*_6,537_ = 3.74, *p *=* *0.001) (Figure 3, Supporting information Figure S1, Table 3). Specifically, we found that pine decline‐tolerant families had significantly greater percentage of colonization by *Thelephora* in Georgia soil compared to pine decline‐susceptible families (*p = *2.0e‐4), but the two categories did not differ in either Mississippi or Alabama soils; fusiform rust‐tolerant and susceptible plants did not differ in *Thelephora* colonization (Figure 3). Pine decline‐tolerant plants had the highest *Thelephora* colonization in Georgia soils (0.31 ± 0.04), which was greater than that in either Alabama (0.09 ± 0.04, *p *=* *2.0e‐16), or Mississippi (0.10 ± 0.06, *p *=* *0.002) (Supporting information Figure S1). Pine decline‐susceptible plants differed in *Thelephora* colonization between Georgia and Mississippi soil inoculum (GA 0.08 ± 0.04, MS 0.20 ± 0.04; *p *=* *0.04) (Supporting information Figure S1). Fusiform‐susceptible plants had the highest percentage of *Thelephora* colonization in Mississippi soil (0.26 ± 0.04), which was higher than that of plants in Alabama soil (0.15 ± 0.04, *p = *0.03), and marginally greater than plants in Georgia soil (0.16 ± 0.04, *p *=* *0.06) (Supporting information Figure S1). There was no difference in *Thelephora* colonization among soil inoculum sources for fusiform rust‐tolerant plants.

**Table 3:** Results of univariate analysis of variance (type III) with Satterthwaite approximation for degrees of freedom. Values shown are *p* values, with bold font indicating *p *< 0.05

Trait	*Wilcoxina*	*Thelephora*
Category	0.29 (*F*_3,10.34_ = 1.41)	0.27 (*F*_3,13.23_ = 1.45)
Soil	**2.10e‐06** (*F*_2,536.05_ = 13.40)	**0.037** (*F*_2,537.95_ = 3.32)
Cat:Soil	0.14 (*F*_6,535.58_ = 1.63)	**0.0012** (*F*_6,537.60_ = 3.74)

	*Cenococcum*	Tips/cm
Category	0.14 (*F*_3,10.93_ = 2.23)	0.5654 (*F*_3,13.89_ = 0.70)
Soil	**<2e‐16** (*F*_2,536.48_ = 123.10)	**6.08e‐05** (*F*_2,534_ = 9.89)
Cat:Soil	0.20 (*F*_6,536.04_ = 1.44)	0.1353 (*F*_6,534_ = 1.63)

	*Rhizopogon*	RGR
Category	0.83 (*F*_3,546_ = 0.30)	**0.025** (*F*_3,14.45_ = 4.22)
Soil	**1.21e‐08** (*F*_2,546_ = 18.86)	**4.13e‐09** (*F*_2,544_ = 20.00)
Cat:Soil	0.20 (*F*_6,546_ = 1.44)	**0.0020** (*F*_6,544_ = 3.53)

	Root:Shoot	
Category	0.65 (*F*_3,13_ = 0.56)	
Soil	0.64 (*F*_2,535.95_ = 0.44)	
Cat:Soil	0.55 (*F*_6,535.77_ = 0.82)	

Of the remaining dominant fungal colonizers, we found soil inoculation source to be the only significant factor to influence rate of colonization. While all three fungal types were found to colonize plants in all soils, we found that each soil inoculum differentially encouraged growth of a different mycorrhizal fungus (Figure 2), regardless of the Genetic Category of the plant families. Particularly, we found that *Rhizopogon* colonization was highest in Georgia soil (*F*_2,536_ = 18.88, *p = *1.20e‐08), *Cenococcum* colonization was highest in Alabama soil (*F*_2,536_ = 123.10, *p = *<2.0e‐16), and *Wilcoxina* colonization was highest in Mississippi soil (*F*_2,536_ = 13.39, *p = *2.10e‐06) (Figure 2, Table 3).

### 3.3 Question 3: How do other traits (host plant relative growth rate, root:shoot ratio, and number of root tips colonized per cm root) respond to host genetic variation in pathogen tolerance and does this genetic variation depend on origin of the fungal community?

#### 3.3.1 Relative growth rate

**Figure 4:**
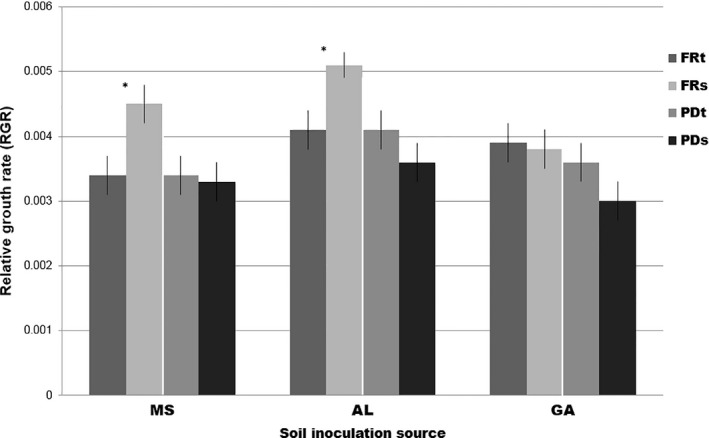
Relative growth rate (RGR) of the four pathogen resistance categories (FRt, fusiform rust tolerant; FRs, fusiform rust susceptible; PDt, pine decline tolerant; PDs, pine decline susceptible), in different soil types (MS, Mississippi; AL, Alabama; and GA, Georgia). The average RGR of seedlings was 2.319 cm (±0.0323 *SE*), and varied according to the interaction between soil inoculation source and the specific pathogen tolerance category of the seedling. Each category of plant family showed a different relationship between RGR and soil inoculation source (Soil × Category interaction: *F*_6,532_ = 3.342, *p = *0.0031). All data are presented as means ± *SE*

The height growth of seedlings averaged 2.32 ± 0.0323 cm total over the 22‐week growing period. The RGR varied according to the interaction between soil inoculation source and the pathogen tolerance Genetic Category of the seedlings (Soil × Genetic Category interaction: *F*_6,544_ = 3.53, *p = *0.002; *R* ^2^m = 0.18, *R* ^2^c = 0.30; Figure 4, Table 3). In both Mississippi and Alabama soils, fusiform rust‐susceptible plants had a higher RGR than fusiform rust‐tolerant plants. There was no difference between fusiform rust‐tolerant or susceptible plants in GA soil, or between plants tolerant or susceptible to pine decline in any of the three soils. Differences between soils, within each Genetic category, are shown in Supporting information Figure S2.

#### 3.3.2 Biomass, root‐shoot ratio, and Tips per centimeter root (tips/cm)

The average dry biomass of the seedlings was 2.84 g, and showed very little variation (±0.004 *SE*). Similarly, we found no significant variation for root–shoot ratio of the seedlings, the average of which was 0.9536 (±0.002 *SE*). On average, loblolly seedlings had 1.02 (±0.02425 *SE*) root tips colonized by EM fungi per centimeter of root length, and did not differ among treatments. Additionally, RGR did not relate to any of the fungal colonization metrics.

## 4 DISCUSSION

The studies presented here lend evidence that several traits of loblolly pine that govern its interaction with members of the fungal community are likely genetically correlated. We found that host plant fungal pathogen tolerance interacted with origin of soil fungal inoculum to influence not only seedling RGR (Figure 4), but also the abundance of a common mycorrhizal fungal morphotype (Figure 3). The variable expression of these traits among different biotic environments (different soil community sources) illustrates the potential for geographic selection mosaics, whereby loblolly pine coevolution with its pathogens may proceed differently in different geographic locations, which can potentially drive trait diversification among populations (Thompson, 2005).

### 4.1 Ectomycorrhizal fungi respond differently to plant pathogen tolerance and soil inoculation source

Across the three soil inoculation sources (MS, AL, GA), four morphotypes of fungi were identified as the most dominant root tip colonizers, *Rhizopogon*,* Cenococcum*,* Wilcoxina*, and *Thelephora*. These are common colonizers of *Pinus* and frequently found on pines in the field (e.g., Hoeksema, Hernandez, Rogers, Mendoza, & Thompson, 2012; Izzo, Nguyen, & Bruns, 2006; Rasmussen, Busby, & Hoeksema, 2018). These results are also consistent with other studies of pines that identify few common species and a large number of rare species (e.g., Izzo et al., 2006; Murata, Kanetani, & Nara, 2017). Of those four, only *Thelephora* was influenced by the genetic category of the host; in contrast, the abundance of the three other dominant mycorrhizal fungal types was found to be influenced by soil inoculum source alone (Figure 2). This result suggests that the host plant, loblolly pine, does not have a single “mycorrhizal interaction” trait, but rather that it interacts with individual fungal taxa, or groups of fungi, in different ways; this has implications for our understanding of the coevolution of these diverse mutualisms, as individual fungi or groups of fungi could be independently exerting selective pressure on different plant traits (Hoeksema et al., 2012). Although studies have shown varying patterns of EM fungal colonization on different age classes of host plants (e.g., Twieg, Durall, and Simard (2007), the importance of mycorrhizal associations in seedling establishment and success is well documented, especially for EM fungi (e.g., Bennett et al., 2017; Booth & Hoeksema, 2010; van der Heijden & Horton, 2009). As such, understanding the genetic basis of seedling interactions with EM fungi is crucial for predicting responses of trees to natural and artificial selection.

This study adds to existing work providing evidence that pines do not have a single “mycorrhizal trait”, but may be evolving independently with different EM fungal species, rather than (or in addition to) the EM fungal community as a whole. Consequently, such diverse mutualisms are not necessarily governed by “diffuse coevolution” in which coevolution is weakened by conflicting selection on the same trait (Hoeksema, 2010). For example, Hoeksema et al. (2012) found that native Monterey pine (*Pinus radiata* D. Don) populations showed genetic variation in patterns of compatibility with different members of the dominant EM fungal community. Specifically, it was found that patterns of genetic variation in compatibility among Monterey pine populations differed among three species of EM fungi: *Rhizopogon roseolus*, Wilcoxina1, and Pyronemataceae1 (Hoeksema et al., 2012).

### 4.2 Potential for selection mosaics on loblolly pine traits

We found the interaction of soil inoculation source and plant pathogen tolerance genetic category to influence abundance of *Thelephora*, and seedling RGR (Figures 3 and 4). It is notable that these patterns were detected in the absence of infection by the fungal pathogens, strongly suggesting a pleiotropic effect of particular genes on these traits and pathogen tolerance, rather than an indirect effect of fungal pathogen infection on both host plant growth and affinity with certain mycorrhizal fungi. Accordingly, natural or artificial selection on pathogen tolerance traits would be predicted to lead to indirect selection on those other two traits, which could reinforce or conflict with direct selection on those traits.

The influence of loblolly pine genetic variation for pathogen tolerance on the abundance of *Thelephora* varied depending on the soil environment, suggesting the potential for a selection mosaic (Thompson, 2005), whereby loblolly pine coevolution with its pathogens may proceed differently in different geographic locations, potentially driving trait diversification among populations. Despite the relatively limited scope of the geographic expanse sampled, we nonetheless found that among the plant tolerance categories, pine decline‐tolerant families especially showed variation in colonization by *Thelephora* among the different soil types (Figure 3). These results suggest a trait‐mediated indirect interaction, whereby the outcome of environmental influence on the loblolly–EM fungal relationship (G × G × E) is mediated by a genetic correlation to pathogen resistance (Ridenhour & Nuismer, 2012). These results predict that if natural or artificial selection favors loblolly pine genetic families that are better able to survive infection by the pathogenic fungi *Leptographium* and *Grosmannia*, we will consequently see increased variation across environments in loblolly pine association with the common fungal symbiont *Thelephora*. Depending on the nature of the interaction between loblolly pine and *Thelephora* in the varying environments (i.e., where it may fall along the mutualism‐parasitism spectrum), there could be conflicts arising from selection pressure for fungal pathogen tolerance and the need for beneficial root symbionts. A similar scenario would be predicted with RGR, an important adaptive trait of the host plant that was also found to vary according to an interaction between plant genetic category and biotic environment. A more thorough exploration of site variation would be needed in order to strengthen the value of these predictions; however, the results shown here do provide an alluring avenue for future studies in the system. It is worth noting that pine decline‐tolerant plants had greater colonization by *Thelephora* than susceptible plants in Georgia inoculated soils (Figure 3), but did not show a greater RGR (Figure 4). This observation may seem to run counter to our general understanding that association with mycorrhizal fungi increases plant fitness, most often indicated by a growth metric. However, the nuances of the symbiosis are revealed by studies on the importance of context‐dependency (e.g., Hoeksema et al., 2010), and other fitness benefits such as drought tolerance (Sebastiana et al., 2018); as such, growth benefits in short‐term experiments may not always be expected, even in symbioses that are beneficial in situ.

Selection mosaics have been explored in previous work in several systems, including plants and mycorrhizal fungi. Piculell, Hoeksema, and Thompson (2008) demonstrated the potential for selection mosaics in the interaction between plants and mycorrhizal fungi by evaluating plant and fungal performance in reciprocal combinations of plant genotypes and fungal genotypes, across two environmental gradients. It was found that both the host plant (bishop pine, *Pinus muricata*) and fungi (*Rhizopogon occidentalis*) had components of fitness that depended on the interaction between the genotype of the associated partner and the abiotic environment (Piculell et al., 2008). Johnson, Wilson, Bowker, Wilson, and Miller (2010) found that the formation of arbuscules (the site of resource exchange between arbuscular mycorrhizal fungi and host plant) by arbuscular mycorrhizal fungi on *Andropogon gerardii* was greater when plants were paired with local mycorrhizal fungi and soil, compared to novel combinations. This result can be explained by a selection mosaic driving local adaptation of plant and fungi to each other, differently in different soils. These experiments, along with others, provide a compelling foundation for the study of geographically driven variation in the outcome of mycorrhizal symbioses. This phenomenon is not, however, limited to these symbioses. Evidence for selection mosaics has been detected across a broad range of interacting organisms. For example, Benkman, Holimon, and Smith (2001) found that the degree of coevolutionary selection between Rocky Mountain lodgepole pine (*Pinus contorta* ssp. latifolia) and red crossbills (*Loxia curvirostra* complex) differed depending on the presence or absence of a third species and superior seed competitor, the red squirrel (*Tamiasciurus hudsonicus*) (Benkman, 1999; Benkman et al., 2001; Thompson, 2005). Similarly, Frederickson et al. (2012) found that the outcome of the interaction between the myrmecophytic *Cordia nodosa* (ant plant), and its resident ant community ranged from beneficial to costly for the host plant, depending on the presence of herbivores. While the work described here focuses on the interaction of pines and mycorrhizal fungi, the findings add to a broader body of work aimed at understanding the diversity and geographic structure of potentially coevolving species interactions.

### 4.3 Geographic variation in EM fungal community of loblolly pine

The abundance of three of the four dominant EM morphotypes, *Rhizopogon*,* Cenococcum*, and *Wilcoxina* was influenced solely by soil inoculation source. Loblolly pine occurs in a large, continuous population across its range with little genetic structure due to high gene flow via widespread windborne pollen (Eckert et al., 2010; Hamrick & Godt, 1996). Given that pine pollen is capable of travelling very long distances and still remains viable (Williams, 2010), the biotic environment where offspring germinate may often be dissimilar to that of both parents, potentially favoring a generalist strategy in the plant hosts regarding EM associations; this may explain why we see no genetic variation for compatibility with three of the four major fungal types found in this study. This result is in contrast to other, more fragmented pine species, such as Monterey pine, which occurs in discrete geographic populations, which have exhibited genetic divergence in their compatibility with several dominant EM fungal species (Hoeksema et al., 2010).

The pattern found in this system, of similar dominant fungi present at all three sites assayed, is consistent with our knowledge of EM fungal communities. A recent assessment of the structure of EM spore community composition on pines in North America found the community was much more structured by geographic region compared with other factors such as climate or host plant identity (Glassman et al., 2015). The proximities of soil locations examined in this study (MS‐AL 313.1 km, MS‐GA 344.9 km, AL‐GA 279.0 km) fall well within the 500 km distance within which Glassman et al. (2015) found fungal spore community similarity to be greatest.

### 4.4 Consequences of trait correlations

Extensive breeding programs, aided with new genomic technologies, have been instituted in an effort to produce stands of fusiform rust‐tolerant trees. This selective breeding process has focused intensely on reducing the susceptibility of tree genotypes to fusiform rust, but there may be unrealized consequences to rust tolerance in the form of unintended indirect genetic effects on other traits. As demonstrated here, the potential exists for correlations between traits driven by genetic mechanisms. Consequently, selective pressure in favor of more pathogen‐tolerant loblolly pine genotypes could impact the performance of these trees in very relevant ways, both ecologically and economically. The results reported here echo those of other studies on the consequences of selection for single traits. For example, a recent study utilizing plant families resistant or susceptible to white pine blister rust (WPBR), a disease caused by the pathogenic fungus *Cronartium ribicola*, found that limber pine (*Pinus flexilis*) seedlings grown from open‐pollinated seed trees known to be either resistant or susceptible to WPBR showed differences in stress tolerance traits, whether or not they had been exposed to the fungal pathogen (Vogan & Schoettle, 2015). Similarly, in a study examining the impact of over 60 years of selective breeding for higher yield in soya beans, Kiers, Hutton, and Denison (2007) found that older soya bean genotypes had higher fitness (measured as seed production) compared to newer genotypes, when exposed to a rhizobia inoculum containing both effective and ineffective strains of the N fixing bacteria. The more recent cultivars were less able to defend against ineffective rhizobia strains, illustrating an unintended consequence of a selective breeding process focused on plant yield. Similar patterns can be expected as a result of natural selection, where strong selective pressure on one trait may facilitate adaptation (or maladaptation) of genetically correlated traits. In the loblolly pine population, for example, strong selection for fungal pathogen tolerance may result in maladapted host plant genotypes in terms of mycorrhizal fungal associations, depending on the soil environment. While seedlings were found to associate with the same dominant fungal types despite soil inoculation source, variation among fungal species in growth strategy and nutrient acquisition abilities makes the difference in relative abundance potentially important.

When selective breeding focuses on one trait, such as pathogen tolerance and growth, there may also be indirect selection on genetically correlated traits. It is important, therefore, to understand the genetic architecture of essential traits in these focal species to better anticipate and manage the results of breeding practices. This study joins others in lending insight into the underlying genetic structure of traits governing species interactions, and how that genetic structure may affect the evolution and adaptation of species. In addition, understanding the genomic architecture of how important adaptive traits are related to one another is an essential component of successful breeding and management strategies.

## CONFLICT OF INTEREST

None declared.

## AUTHOR CONTRIBUTIONS

B.J. Piculell, L.G. Eckhardt, and J.D. Hoeksema conceived and designed the experiments. B.J. Piculell implemented the experiments, collected, and analyzed data with assistance from J.D. Hoeksema. B.J. Piculell authored the manuscript, with editorial comments from L.G. Eckhardt and J.D. Hoeksema.

## DATA ACCESSIBILITY

Mycorrhizal colonization data have been archived as supporting information on Dryad (https://doi.org/10.5061/dryad.n13h839).
