# Supplementary material for: Genetically determined fungal pathogen tolerance and soil variation influence ectomycorrhizal traits of loblolly pine
Source: Ecol Evol. 2018 Sep 5;8(19):9646–56. doi: 10.1002/ece3.4355 (PMC6202710; doi:10.1002/ece3.4355)
Supplement: Supplementary file 1 [file ECE3-8-9646-s001.docx]

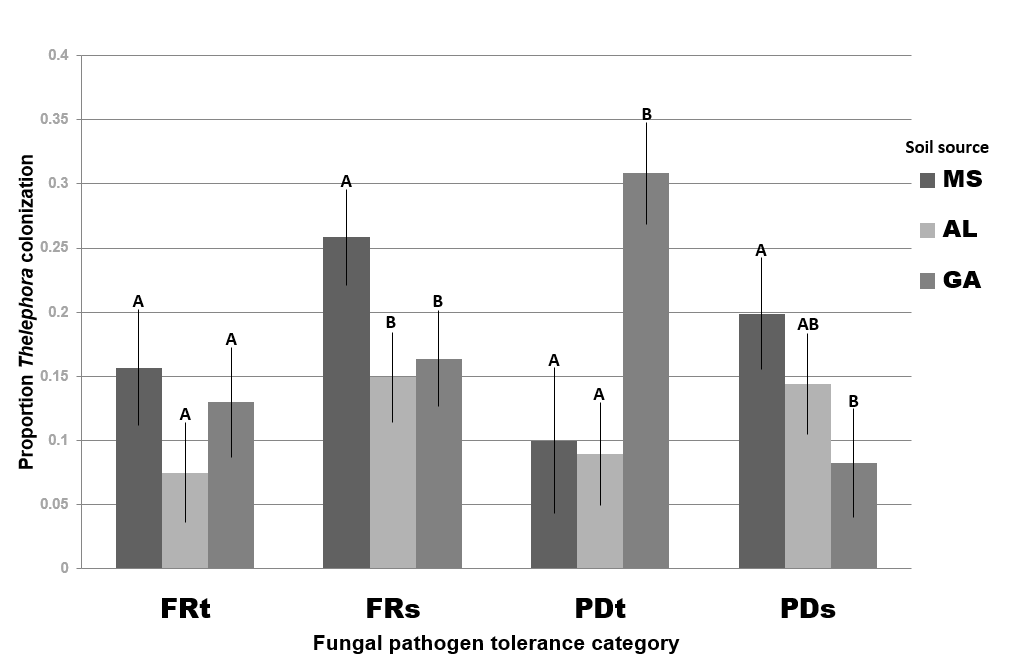


**Supplementary figure 1:** The influence of pathogen resistance category (FRt, Fusiform rust tolerant; FRs, Fusiform rust susceptible; PDt, Pine decline tolerant; PDs, Pine decline susceptible) on proportion of root tips colonized by *Thelephora* varied among soil inoculation sources (MS, Mississippi; AL, Alabama; and GA, Georgia), (Category x Soil interaction: F_6,537_ = 3.74, *p* = 0.001, Table 3). All data are presented as means ± SE. Tukey posthoc letters indicate differences between soil inoculation sources, within plant genetic category.


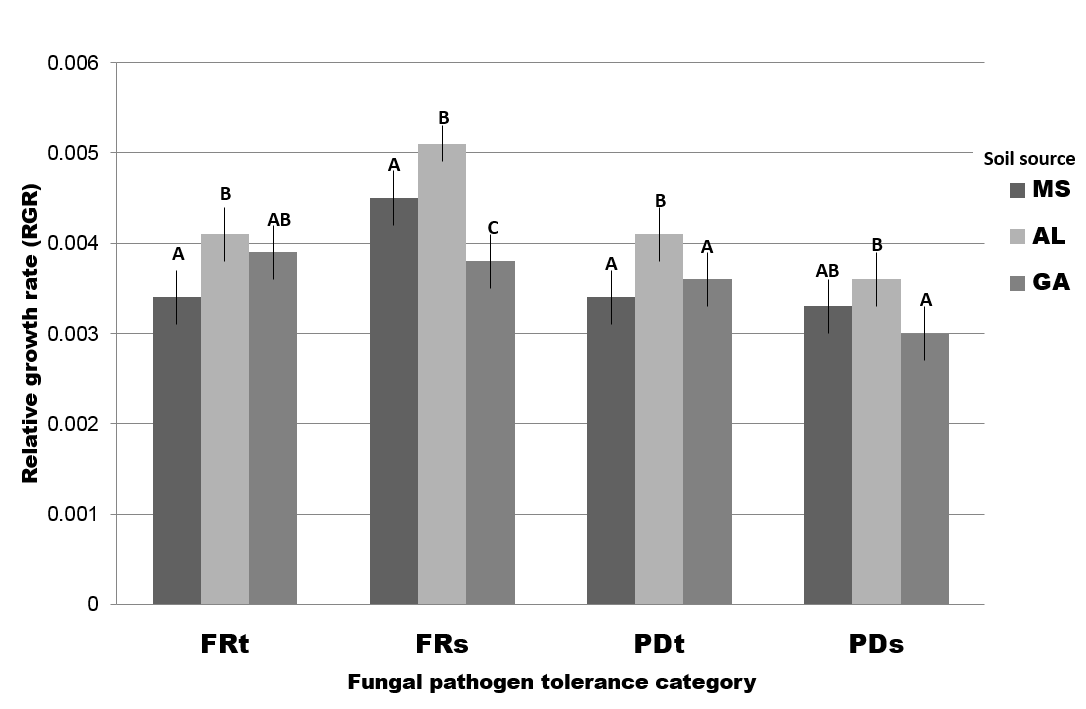


**Supplementary figure 2:** Relative growth rate (RGR) of the four pathogen resistance categories (FRt, Fusiform rust tolerant; FRs, Fusiform rust susceptible; PDt, Pine decline tolerant; PDs, Pine decline susceptible), in different soil types (MS, Mississippi; AL, Alabama; and GA, Georgia). The average RGR of seedlings was 2.319 cm (± 0.0323SE), and varied according to the interaction between soil inoculation source and the specific pathogen tolerance category of the seedling (Table 3). Each category of plant family showed a different relationship between RGR and soil inoculation source (Soil x Category interaction: F_6, 532_ = 3.342, *p =* 0.0031). All data are presented as means ± SE. Tukey posthoc letters indicate differences between soil inoculation sources, within plant genetic category.
